# Baltic dry index forecast using financial market data: Machine learning methods and SHAP explanations

**DOI:** 10.1371/journal.pone.0325106

**Published:** 2025-07-21

**Authors:** Hyeon-Seok Kim, Do-Hyeon Kim, Sun-Yong Choi

**Affiliations:** 1 Department of Industrial Engineering, Hanyang University, Seoul, Republic of Korea; 2 Department of Finance and Big Data, Gachon University, Seongnam, Republic of Korea; Shanghai Jiao Tong University, CHINA

## Abstract

The Baltic Dry Index (BDI) is a critical benchmark for assessing freight rates and chartering activity in the global shipping market. This study forecasts the BDI using diverse financial data, including commodities, currencies, stock markets, and volatility indices. Unlike previous research, our approach integrates financial indicators specific to major marine trading regions—the U.S., EU, and Hong Kong. We employ advanced machine learning methods, such as Extremely Randomized Trees, Categorical Boosting (CatBoost), and Random Forest, to achieve superior forecasting accuracy. Additionally, we utilize the Shapley Additive Explanations (SHAP) framework to analyze the contributions of financial features to BDI predictions. Key findings reveal that the S&P 500 index is the most influential factor, followed by significant contributions from iron ore and coal commodity indices and the dollar index, underscoring the interplay between the U.S. economy and the BDI. By integrating SHAP explanations, this study not only predicts market trends but also uncovers the economic drivers shaping the BDI. Practically, it supports the stability of the global shipping industry by enabling more informed decision-making for stakeholders. Academically, it introduces overlooked economic factors in BDI prediction, offering valuable insights and directions for future research.

## Introduction

BDI is a key indicator that measures global shipping rates for transporting dry bulk commodities, such as coal, iron ore, and grain. It is compiled daily by the Baltic Exchange in London and reflects the cost of moving raw materials by sea. The BDI is composed of three sub-indices, each tracking different sizes of bulk carriers: Capesize, Panamax, and Supramax.

The most important element in the BDI is dry bulk, which mainly consists of materials used in the production of intermediate goods or finished products, such as steel, food, and concrete. Owing to its purpose, dry bulk can be an efficient economic indicator of future production and growth in the industry. Therefore, BDI, which represents the volume of dry cargo, can be considered a leading economic indicator, and many studies have attempted to forecast BDI or find its relationship with other significant economic indicators.

Forecasting the BDI is crucial not only for stakeholders in the marine transport industry but also for participants in the broader global economy. This study aims to predict the BDI using diverse economic data and advanced machine learning techniques known for their exceptional predictive performance. By doing so, we seek to deliver highly accurate forecasts, providing valuable insights for industry leaders and economic decision-makers.

Several studies have investigated the BDI’s connection with economic indicators, revealing that the BDI is more than a mere shipping trade measure and is linked to various economic metrics. [1] explained the role of BDI in predicting the future course of the real economy, establishing a link between financial asset markets and the macroeconomy with the behavior of financial asset prices and industrial production. [2] affirmed that the expansion of trading in less developed countries resulted in a positive relation between income and living standards, as demonstrated through experiments with BDI. [3] investigated the spillover effects of BDI depending on the sample period used in the article, and the spillover effect on the price volatility of shipping stocks, the United States (US) currency market, and the commodity futures market. These articles recognized BDI as a significant indicator of the global economic sector.

As BDI is crucial, forecasting it is important to the industry and researchers. The importance of forecasting BDI can be explained from various perspectives. From a logistical standpoint, especially in shipping, vessels handle more than 80% of international trade (IMO, https://imo.libguides.com/MaritimeFactsandFigures). Additionally, over half of the shipping industry involves carrying dry bulk freight and other dry goods (“World seaborne trade”, https://hbs.unctad.org/world-seaborne-trade). Therefore, forecasting BDI can assist business and management decisions made by executives of marine transport companies. It can help them deal with potential crises in the shipping industry and make decisions to maximize their revenue based on the movements of BDI. From an economic perspective, the shipping industry can be sensitive to financial issues or global phenomena. For instance, the global financial crisis in 2009 and the prohibition of liner conferences by the European Union (EU) had respective effects on the shipping industry. Whether indirectly or directly, BDI can fluctuate in response to such issues, and this is where forecasting BDI by identifying the detailed factors influencing it becomes crucial. Consequently, a substantial body of research has focused on predicting this BDI. A comprehensive review of relevant literature is provided in Literature Review section.

Methodologically, we employ multiple machine learning methods to predict the BDI, including Random Forest (RF), Extreme Gradient Boosting (XGBoost), Categorical Boosting (CatBoost), Extremely Randomized Trees (Extra Trees), and Light Gradient Boosting Machine (LightGBM). These models are briefly reviewed in section Methods. To enhance our forecasting features, we utilize financial datasets consisting of commodity prices, crude oil prices, the dollar index (DXY), and stock market indices from three countries. In the following literature review section, we provide an analysis of prior studies that examine the relationship between these indicators and the BDI.

To investigate the contribution of each parameter in forecasting the BDI, we use Shapley additive explanations (SHAP) developed by [4]. The SHAP framework allows us to enhance the accuracy and reliability of our predictions by providing insights into the significance of each variable.

Overall, our research workflow involves determining the contribution of various financial factors in forecasting the BDI using a range of machine learning algorithms. This approach strengthens the robustness of our prediction models, validates the effectiveness of our machine learning algorithms, and provides insights into the key drivers of the BDI.

This study sets itself apart from previous research on BDI prediction by incorporating financial data and indices specific to the most influential marine trading regions: the U.S., EU, and Hong Kong. Unlike traditional approaches that focus solely on tracking the BDI through methodological advancements, our approach leverages selected financial indicators to capture the relationships between the BDI and key trade hubs. This focus underscores both the novelty and the practical applications of our findings, providing industry stakeholders with a specialized tool for more informed decision-making.

This study offers several significant and novel contributions to the field of maritime economics and freight market forecasting. Unlike existing literature that predominantly relies on conventional econometric models or limited variable sets, this research distinguishes itself by integrating advanced machine learning techniques to achieve notably higher predictive accuracy for BDI. This methodological innovation not only enhances forecasting performance but also represents a departure from traditional approaches commonly used in the domain. Furthermore, the study expands the scope of input variables by incorporating a diverse range of financial and macroeconomic indicators, many of which have been overlooked in previous BDI-related research. This broader variable inclusion allows for a more comprehensive assessment of the factors driving freight market dynamics. Most importantly, the application of the SHAP algorithm introduces a transparent and interpretable framework to identify the most influential predictors, a feature rarely explored in existing maritime forecasting studies. These innovations collectively deepen both the theoretical and practical understanding of global shipping markets. By bridging methodological advancement with real-world applicability, the study provides actionable insights for industry practitioners, financial analysts, and policymakers engaged in freight risk management and maritime economic strategy.

Previous studies have analyzed the performance of econometric models, ANN, and hybrid approaches for BDI forecasting. [7] compared ARIMA and GARCH with BPNN, RBFNN, and ELM. While GARCH performed better than ARIMA in short-term forecasts, ANN models showed lower errors in weekly and monthly predictions. During high market volatility, such as the 2008 crisis, GARCH had larger forecast errors than ANN, suggesting limitations in handling extreme conditions.

[10] showed that the ARIMA model had significantly higher RMSE than deep learning models, with short-term prediction errors approximately two to three times higher and long-term errors increasing substantially, in some cases exceeding seven times the error of deep learning models. These findings suggest that traditional econometric models, while useful for short-term predictions, struggle to capture the nonlinear and dynamic patterns in BDI data.

This study employs machine learning ensemble models, such as Random Forest and CatBoost, to enhance both predictive accuracy and interpretability. Unlike ARIMA and GARCH models, which assume stationarity and linearity, these ensemble methods effectively capture nonlinear interactions between financial and macroeconomic indicators. Compared to ANN-based models, which often function as black boxes, the use of SHAP analysis offers clearer insights into key variables such as the S&P 500 and DXY. By incorporating a broader set of financial and economic indicators, this approach improves predictive performance across multiple time horizons while also enhancing its practical applicability in the shipping and financial sectors.

Our study has significant implications both practically and academically. Practically, it contributes to enhancing the stability of the global shipping industry by providing a sophisticated model for predicting the BDI. This model helps mitigate risks associated with the uncertainty of the shipping market’s future, enabling stakeholders to develop more effective strategies to address potential challenges. Academically, our study introduces a range of economic factors influencing BDI predictions that have been insufficiently considered in previous research. By highlighting these factors, our work opens new avenues for studies on BDI prediction and related topics, offering valuable insights to the academic field.

The remainder of the paper is organized as follows. In the following section, we review the literature on BDI, categorizing it into studies focused on predicting BDI using different methods, and those examining the connection between BDI and economic indicators. Data Description section describes the data and provides a preliminary BDI statistical analysis and the employed data. In Method section, we explain the research design and machine learning methods. Section Results and discussion section presents the forecasting results and discussion. Finally, we provide the summary and concluding remarks in the last section.

## Literature review

In this section, we introduce the literature on BDI, dividing it into BDI-prediction studies using various methodologies and studies on the relationship between BDI and economic indicators.

### Forecasting BDI by using various models

Many studies applied various types of machine learning models to predict BDI.

[5] applied the support vector machine model with correlation-based feature selection to predict the monthly movement of the BDI. They selected the correlation-feature-selection method to reduce the calculation by decreasing model duplication. The authors focused on finding the relationship between freight and BDI to support decision making for participants in the industry. They found that the support-vector-machine methods accurately and reliably predicted the BDI index.

[6] adopted the empirical mode decomposition (EMD) into an ANN to forecast BDI. They added a composition mode to improve the performance of the EMD with ANN (EMD-ANN) model. This EMD-ANN model efficiently handles the nonlinear and non-stationary BDI characteristics using an integrated model. In their search for better forecasting results, they used the composition procedure known as error correction EMD with ANN. However, the composition work did not improve prediction.

[7] examined the accuracy of univariate econometric models, specifically ARIMA and GARCH, and compared them with three ANN-based algorithms: Back Propagation Neural Network (BPNN), Radial Basis Function Neural Network (RBFNN), and Extreme Learning Machine (ELM). The key findings from studies: First, econometric models produce better one-step-ahead daily predictions than ANN-based algorithms. However, for weekly and monthly data predictions, ANN algorithms exhibit superior performance by generating fewer errors and a higher direction matching rate. Second, Econometric models are more suited for short-term daily predictions, while ANN models are more effective for forecasting over longer time scales and multiple periods ahead.

[8] introduced five technical indicators (%R, relative strength index, moving average convergence divergence, commodity channel index, moving average) and used a fuzzy neural network to forecast BDI. They obtained better returns compared to those of other forecasting methods. The authors remarked that accurate forecasting can assist ship owners in making various business decisions based on short- or long-term predictions.

[9] proposed a dynamic fluctuation network to capture the non-linear and non-stationary BDI characteristics. This network combined three ANN models: back propagation (BP), radial basis function (RBF), and extreme learning machine (ELM). The purpose was to compare the network with the original BP, RBF, and ELM models in predicting BDI. The authors found that these novel models were unaffected by training and test sample selection, and were robust with regard to data frequency and extreme market switches. Thus, the proposed models are suitable for making chartering and ship-building decisions under uncertainty.

[10] proposed an integrated deep-sequential model called deep ensemble learning (DERN) model, composed of the recurrent neural network (RNN), long-short-term memory (LSTM) network, and gated rectified unit neural network (GRU). The authors compared the DERN model to previous methods such as ARIMA, multi-layer perceptron (MLP), LSTM, GRU, and RNN to assess the performance difference. The DERN model showed the best prediction results and lowest error rates in short-term predictions. However, its performance was not as good in long-term predictions compared to short-term predictions.

[11] used 42 potential BDI influencing factors, such as vessel order book by size, vessel contracting by size, world seaborne trade index, and commodity prices, which are macroeconomic indicators. These factors were applied in a deep-learning framework to enhance forecasting accuracy. The inclusion of these factors improved accuracy, and the deep-learning framework outperformed previous models, including RF and support vector regressor, by extracting multiple levels of representations from the features. They mentioned that incorporating more potential factors can lead to even more effective predictions.

As outlined earlier, numerous methodologies have been proposed for predicting the BDI, each with its own set of strengths and weaknesses. SVM-based models effectively capture nonlinear trends and integrate macroeconomic indicators, but their reliance on dataset-specific parameter tuning limits adaptability [5]. EMD combined with neural networks handles nonlinear and non-stationary data robustly, though its complexity hampers modeling diverse frequency components [6]. Econometric models outperform ANN models in daily predictions, while ANNs excel in weekly and monthly forecasts, but hybridization to leverage both remains limited [7]. Fuzzy neural networks with technical indicators achieve high accuracy (83%) but struggle during volatile periods [8]. Dynamic Fluctuation Networks (DFN) address abrupt changes effectively but are prone to parameter sensitivity and overfitting [9]. The DERN deep ensemble model (RNN, LSTM, GRU) shows superior predictive capabilities but lacks detailed analysis of ensemble effectiveness [10]. Finally, deep learning frameworks combining feature extraction and regression improve prediction accuracy but face challenges with limited features and runtime optimization [11].

These studies demonstrate various strengths and accomplishments across different methodologies, but issues such as model complexity, sensitivity to parameter adjustments, and applicability limitations suggest that optimization is necessary under specific conditions.

### The relationship between BDI and economic indicators and financial assets

The relationship between BDI, economic indicators, and financial assets has been analyzed in several studies. [1] demonstrated the relevance of BDI as an indicator that captures the relationship between the financial asset market and the macroeconomy. To prove the usefulness of the BDI, panel cointegration tests were conducted using financial and industrial indicators such as the annual price (AP) of stock market returns (APSTK), AP interest rates on short-term bonds (APSTB), AP interest rates on long-term bonds (APLTB), AP commodity prices (APCOM), and oil prices from G7 countries (Canada, France, Germany, Italy, Japan, U.K.). The results supported the relationship between BDI and the real economy as the demand and production of commodities were influenced by the movement of BDI.

[12] introduced a technical indicator called the dry bulk economic climate index (DBECI), which is composed of eight sub-indicators, including Euro/USD exchange rates, Yuan/USD exchange rates, Brent crude oil price, and the Federal funds rate in the US. This index was introduced to enhance the robustness and forecasting accuracy of BDI. Two different modeling approaches, ARIMA and VARX models, were used to predict BDI. Results showed an impressive relationship between BDI prediction and DBECI, further reinforcing the robustness and accuracy of BDI prediction.

[13] provided novel results regarding BDI that can be used for investment exercises and improving risk management in the shipping industry. They set the prices of eight major commodities, such as wheat, coal, and iron ore, as well as financial indicators, including crude oil prices, Morgan Stanley global indices for emerging market and developed market, the British pound/US dollar exchange rate (GBP/USD), the dollar index (DXY), and the 10- and 2-year US Treasury yield difference (SPREAD). These factors were investigated using a regression model to identify explanatory factors in the cyclical nature of BDI. They found a strong cyclical pattern that highly fluctuated between 3 and 5 years, and this pattern remained stable across the period. Furthermore, these indicators supported the forecasting of future BDI trends but were not suitable in every situation.

[14] pointed out the causality between BDI and exchange rates. Using panel and individual time-series regression, they found some significant results. They found BDI to have long-term predictability against exchange rates, and the returns of exchange rates had a negative correlation with changes in BDI. BDI exhibited an inverted U-shaped pattern that peaked at a 1-year forecast horizon, implying that BDI could provide information for predicting exchange rates.

[15] delved into how the COVID-19 pandemic influenced maritime shipping freight rates through an asymmetric Multifractality Detrended Fluctuation Analysis (A-MFDFA). They found multifractal behaviors in major maritime shipping freight indices, with a pronounced increase in multifractality correlating with the fractal scale. Specifically, the research highlighted that during the pandemic, the Baltic Dry Index (BDI) and the Baltic Clean Tanker Index (BCTI) exhibited greater multifractality in downward movements, indicating reduced efficiency and predictability. Conversely, the Baltic Dirty Tanker Index (BDTI) demonstrated increased multifractality in upward movements during the same period. This asymmetric response suggested that the maritime shipping freight market has become inefficient, especially during the pandemic, necessitating stringent monitoring and regulation to manage the volatility and unpredictability in freight rates effectively.

[16] utilized a nonparametric causality-in-quantiles (CiQ) approach, the study identified the existence of asymmetric and nonlinear causality between the BDI and commodity prices across different market conditions (bearish, normal, bullish). The study observed that commodity prices significantly influence the BDI across all market conditions, with the strongest effects noted during normal periods. Conversely, the impact of the BDI on commodity prices varies considerably across the range of commodities and market conditions. The study also concluded that commodity prices are a leading indicator for the BDI, providing valuable insights for commodity market participants to effectively hedge against variations in commodity prices and freight rates.

Building upon prior research, this study leverages a recently popular machine learning approach to predict the BDI. Furthermore, we incorporate a diverse set of economic and financial indicators into the prediction model. To distinguish our work from previous studies, we employ the SHAP framework to quantify the individual contribution of each factor to the BDI prediction.

The BDI is broadly validated in the reviewed studies as a key predictor of economic growth and business cycle patterns. For example, [12] highlight its strong connection to dry bulk shipping and economic activity in China, particularly steel production, reflecting broader economic conditions. A rising BDI signals economic recovery and increased raw material demand. Similarly, [15] note the significant drop in the BDI during the COVID-19 pandemic, indicating reduced raw material demand, manufacturing slowdowns, and economic contraction. The BDI is also a valuable predictor for financial assets, including exchange rates and commodity prices. [14] demonstrate its long-term influence on exchange rate fluctuations, while [16] reveal its asymmetric relationship with commodity prices, underscoring its utility in forecasting market trends.

However, interpretations of the BDI’s predictive cycle and its industry-specific impacts vary across studies. [13] find the BDI effective for long-term forecasts linked to economic cycles, while [15] emphasize the stronger influence of short-term fluctuations during events like COVID-19, suggesting its predictive power depends on context. Industry-specific impacts also differ; [12] highlight its strong link to Chinese steel production, whereas [16] focus on its association with the commodities market. Furthermore, sub-indices of the BDI, such as Capesize, Panamax, and Supramax, may offer greater predictive accuracy for specific variables, as suggested by [13].

Based on the above discussion, we find a close relationship between the BDI and the financial market. Previous studies have explored the connections between the BDI and various components of the financial market, including commodities [13,16], currencies [12,14], and stock markets [1,13]. Accordingly, we propose the following hypothesis.

**Hypothesis 1**. Commodities, currencies, and stock markets exert significant influence on BDI predictions.

Existing research has utilized various commodity assets, some of which are directly related to the calculation of the BDI. Based on this, we propose the second hypothesis.

**Hypothesis 2**. The influence of commodity assets on BDI predictions varies depending on the type of asset.

Similarly, regarding currencies, we suggest the following hypothesis.

**Hypothesis 3**. The influence of individual currencies on BDI predictions differs based on the specific currency.

Finally, drawing on previous findings that the economic conditions of individual countries affect the BDI [12], we propose the last hypothesis.

**Hypothesis 4**. The stock markets of individual countries exert varying degrees of influence on BDI predictions.

Methodologically, this study employs a recently popular machine learning approach to predict the BDI. Machine learning methods are known to outperform traditional methodologies in terms of predictive accuracy [17–19]. By utilizing these advanced techniques, this study aims to achieve superior predictive performance compared to previous research.

In addition, the study incorporates the SHAP framework to evaluate the contribution of individual factors in predicting the BDI. This enables the validation of the previously proposed hypotheses by identifying the relative importance of various financial indicators. Unlike prior studies that primarily focused on prediction, this approach provides evidence on which financial indicators are most critical for BDI prediction, offering deeper insights into the factors influencing the shipping market.

## Data description

In our study, we used a diverse range of financial data classes, including commodities, foreign exchange, stock indices, and volatility, to forecast BDI. The selection of commodities, currencies, stock indices, and volatility as financial data for predicting the BDI is based on their direct and indirect influence on global trade and shipping demand. Moreover, within each of these classes, we incorporated multiple datasets for a comprehensive analysis. First, we used the prices of four commodities: iron ore, coal, and wheat, as well as crude oil (Brent Europe, WTI). Commodities, such as iron ore, coal, and grain, are key cargoes transported by dry bulk ships, making their price fluctuations a crucial determinant of freight rates. Generally, studies that explore the relationship between commodities and BDI have used eight representative commodities, such as iron ore, aluminum, coal, copper, soybean, and wheat [13,16]. [16] found that iron ore, coal, and wheat exhibit bidirectional causality with BDI, regardless of the market trend. According to this study, we assume that these three commodities have a crucial impact on BDI, while other commodities exhibit only unidirectional or insignificant relationships. Additionally, [56] highlight the strong interconnectedness between commodity prices and global financial markets, suggesting that fluctuations in key raw materials significantly influence economic activity and trade volumes. Based on these findings, we included iron ore, coal, and wheat as essential predictors of BDI, while excluding other raw materials, such as copper and gold, due to their weaker direct association with bulk shipping rates.

We also consider crude oil as an important predictor of BDI. [56] demonstrate that oil price shocks have a substantial impact on financial markets, including global stock indices and exchange rates. This reinforces the rationale for incorporating Brent Crude and WTI Crude as key indicators of shipping costs, which directly influence BDI fluctuations. [13] and [12] used crude oil prices (Brent Europe) as an indicator for forecasting BDI. Crude oil has a relationship with BDI [20] and plays a significant role in the shipping industry [21].

We also used the DXY (U.S. Dollar Index) to measure the value of the US dollar relative to a basket of major world currencies, particularly those of major exporting and importing nations, impact trade costs and competitiveness, influencing shipping demand. It serves as a benchmark for the dollar’s performance in international markets and reflects changes in the dollar’s value against these currencies. [55] highlight that US monetary policy has a dominant influence on the global financial cycle, affecting capital flows, risk appetite, and asset prices across markets. Given that DXY reflects global trade competitiveness and financial stress, its inclusion as an economic indicator in our analysis is well justified. [3] found that BDI exhibited volatility spillover effects on DXY, reinforcing its role in capturing macro-financial linkages. Similarly, [13] utilized DXY as an economic sub-indicator to identify cyclical patterns and aid in forecasting the BDI.

EU is one of the active markets in international maritime trade (“world trade statistical review 2022”, https://www.wto.org), we incorporated the euro index and Eurostoxx50 as significant economic variables. The Euro Index is a measure of the value of the euro (EUR) relative to a basket of other major currencies, and provides a snapshot of the euro’s strength or weakness against these currencies.

We also used stock market indices from three different countries and regions: the EU, Hong Kong, and the US These countries and regions rank among the highest in transport-related exports and imports (“World Trade Statistical Review 2022”, https://www.wto.org). Given their major role in global trade, serve as a proxy for overall economic health, as strong equity markets often indicate increased industrial production and trade activity, driving higher shipping demand we included these indices to capture financial market dynamics that could influence BDI.

Moreover, we employed the volatility indices of the three major stock markets, commonly known as fear indices reflect market uncertainty, which can affect investment decisions, global trade flows, and, consequently, freight rates. These indices serve as crucial indicators reflecting the level of uncertainty and risk perceived by market participants in prospective stock market conditions. Consequently, we investigated how the negative outlook of financial markets affects BDI predictions using volatility indices such as VIX, VHSI, and VSTOXX.

While other financial and economic indicators, such as Nikkei 225, KOSPI, USD/KRW, and copper prices, could theoretically influence the BDI, they were excluded due to their more localized or redundant nature. [55] emphasize that the global financial cycle is predominantly driven by US monetary policy and major financial markets rather than regional stock indices. Hence, including S&P 500 and DXY provided a more comprehensive representation of global financial trends without the need for region-specific indices. Additionally, [56] suggest that while commodity prices impact financial markets, Brent crude and WTI crude exhibit stronger connections with macroeconomic activity than metals like copper or gold, justifying our focus on oil as a primary energy input affecting shipping costs.

All the data we used was sourced over the period from January 3, 2018, to June 30, 2023, with gaps in the data meticulously removed. The reason for removing some data is due to the gaps in closing prices caused by time differences and public holidays among different countries, involving stocks, commodities, currencies, and volatility indices. We first collected the closing prices of all selected indicators. If any single indicator had a missing closing price on a given date, that date was excluded from the dataset. For instance, if all indicators except the Euro Index had recorded closing prices on December 21, 2019, the data for that date was removed due to the missing value for the Euro Index. As a result, only dates on which all indicators had available closing prices were retained in the final dataset, covering the entire period from January 2018 to June 2023. Plus, the source for all these indices such as commodities, crude oil, currency, stock, volatility, and BDI was Investing.com (source: https://www.investing.com). Notably, while BDI data are gathered daily, it is important to acknowledge that other datasets (excluding the BDI) may exhibit variations in terms of timing and potential discrepancies. Considering these disparities, our approach involved the use of weekly average data intervals for the analysis of these financial metrics.

In particular, this study employs weekly data to better capture market dynamics over shorter time intervals, in contrast to previous studies that primarily utilized monthly BDI data. Since the financial data used in our analysis are available on a daily basis, conducting the analysis on a weekly frequency is both feasible and appropriate for the objectives of this research.

Table 1 presents the average of processed weekly average statistical data. Volatility indices exhibit similar statistical characteristics. Among these, Coal demonstrates the highest volatility as indicated by its standard deviation. Additionally, the BDI also shows significant fluctuations.

**Table 1:** Descriptive statistics of the weekly average data.

Sectors	Asset	Mean	Max.	Min.	Std.Dev.	Skewness	Kurtosis
Commodity	Wheat	640	1224.50	425.63	167.27	1.29	1.46
	Iron Ore	112.33	218.83	63.23	36.40	0.98	0.66
	Coal	124.58	391.77	38.68	93.05	1.48	1.11
	Brent Crude Oil	70.72	122.30	20.49	19.49	0.12	0.25
	Crude Oil WTI	65.69	120.93	8.10	19.57	0.21	0.46
Currency	DXY	97.42	112.85	89.63	5.16	0.81	0.39
	Euro index	104.44	125.36	91.81	8.96	0.36	-0.80
Stock index	HSI	25177.29	31518.66	15092.26	3644.78	-0.53	-0.57
	Eurostoxx50	3637.58	4396.55	2480.89	399.03	0.07	-0.60
	S&P 500	3529.26	4716.80	2405.55	658.91	0.11	-1.43
Volatility index	VIX	21.20	70.40	12.04	7.92	2.40	10.12
	VHSI	23.03	57.10	13.75	5.87	1.61	4.94
	VSTOXX	21.42	74.61	11.19	8.25	2.43	10.22
	BDI	1687.42	5544	411.6	890.06	1.44	2.77

**Fig 1:**
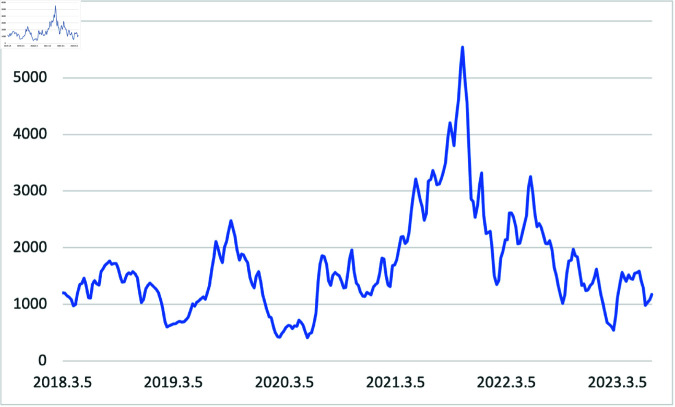
Trend of BDI Index from 01/03/2018 to 30/06/2023, separated by weekly data.

Fig 1 shows the BDI index during the sample period from March 2018 to June 2023. The first quarter of 2019, especially in February and March, hit its lowest point of the year because of the decline in freight charges of Baltic capesize carriers, which were affected by Vale’s dam collapse in Brazil [23]. After the tragic accident in the third quarter of 2019, BDI increased significantly owing to several factors. First, Brazil experienced problems with supplying iron ore. Second, lower Chinese iron ore inventory levels and rising iron ore prices led to increased demand for securing Chinese iron ore. Third, ships entering the port for scrubber installation due to IMO environmental regulations were concentrated in the third quarter. A significant peak was reached in October 2021, shortly after the end of the COVID-19 pandemic. During the pandemic, a shortage of bulk carriers and containers led to mixed freight being transported by small bulk carriers and individual ships. Moreover, the demand for iron ore also increased, which contributed to the highest BDI in 2021.

**Fig 2:**
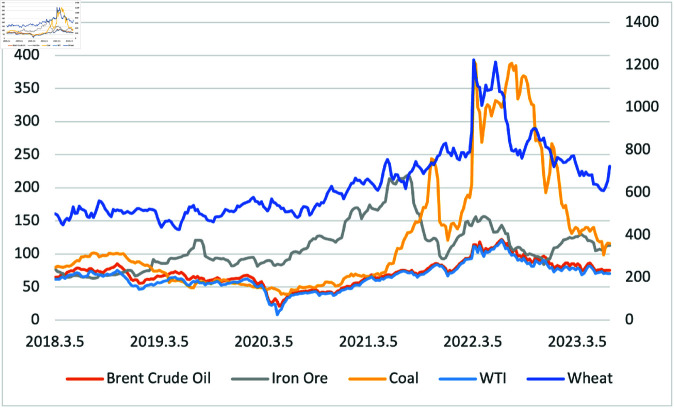
Trend of 5 Commodities price(Wheat, Coal, Crude Oil Brent, Crude Oil WTI, Iron Ore) from 01/03/2018 to 30/06/2023. The right y-axis refers to the Wheat price.

Fig 2 illustrates the prices of five commodities during the mentioned sample period. Owing to the higher price range of wheat compared to others, we included the price range of wheat as a sub y-axis on the right side of the graph. Wheat and coal prices similarly peaked around June 2022, while Brent crude and crude WTI exhibited almost the same movement during the sample period.

**Fig 3:**
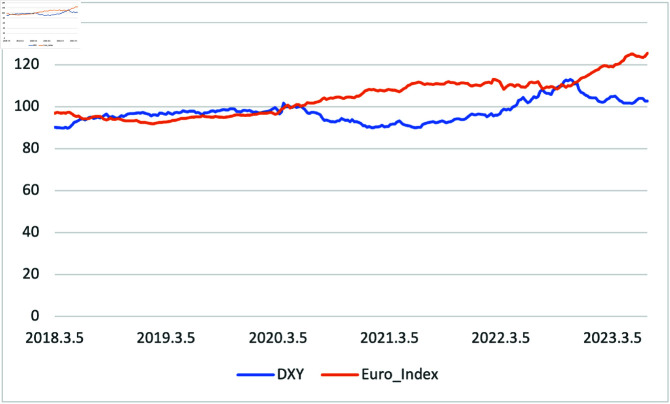
Price of 2 Currencies (U.S. Dollar and Euro) from 01/03/2018 to 30/06/2023, divided by weekly data.

Fig 3 presents the prices of two currencies during the sample period. DXY and the euro showed the most significant gap around June 2021. In general, the euro slightly increased during the sample period, whereas DXY decreased during the COVID-19 pandemic and increased afterward, only to drop again around September 2022.

**Fig 4:**
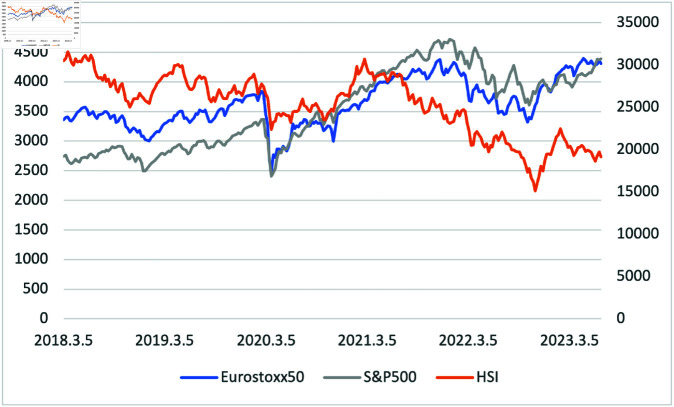
Three Stock Indices (Eurostoxx50, S&P 500, and HSI) from 01/03/2018 to 30/06/2023, The right y-axis refers to the HSI price.

Fig 4 illustrates three stock indices during the sample period. The value of HSI was significantly higher than S&P 500 and Eurostoxx50; therefore, we included the range of HSI as a sub y-axis on the right side of the graph. Regardless of their actual values, all three indices exhibited almost the same movement during the sample period, especially Eurostoxx and S&P 500, which showed similar patterns.

**Fig 5:**
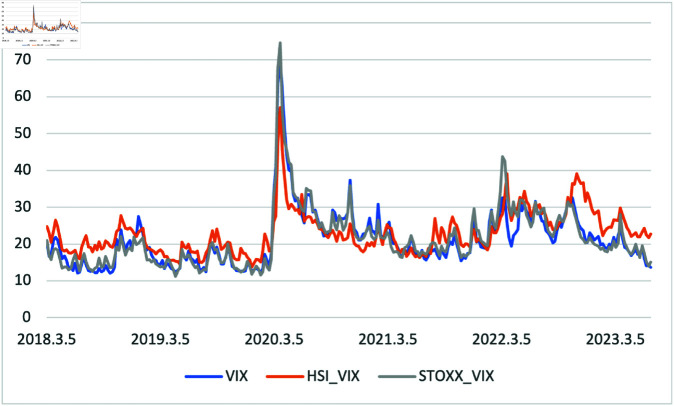
Three respective volatility index from 01/03/2018 to 30/06/2023, divided by weekly data.

Fig 5 illustrates three volatility indices for each of the three stock indices mentioned earlier. Unlike other indices, there are two significant points in this figure. All volatility indices showed the most accurate movement by themselves, and they all peaked around March 2020 during the COVID-19 pandemic.

## Methods

### Research process

We employed several machine learning algorithms to forecast the contribution of economic indicators and dry commodities in predicting BDI. We used price data from five commodities, two currency prices, three stock index data, and three volatility stock index data to predict the contribution of variables to BDI. To assess the impact of commodities and economic variables on the prediction of the BDI, we employed the SHAP framework to evaluate their contributions. In this analysis, we used economic data and commodities prices as benchmarks to compare the respective contributions of variables in forming the BDI index.

**Fig 6:**
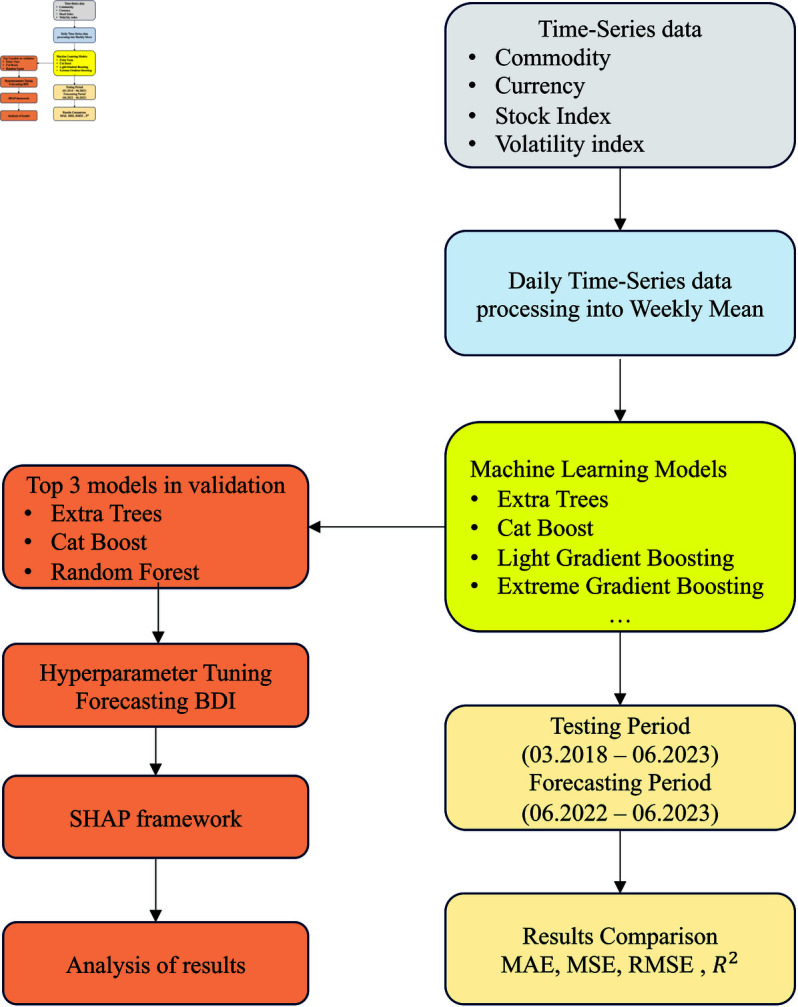
The flowchart of this study.

Furthermore, we employed multiple machine learning algorithms to forecast weekly data. Through an evaluation between the machine learning algorithms, we selected several machine learning algorithms based on forecasting performance. The hyperparameters of the selected models were then optimized to enhance performance. Subsequently, we used the optimized machine learning models to predict BDI. Finally, we applied the SHAP framework to the prediction results, allowing us to evaluate the contribution of these commodities in the forecasting process. This workflow is shown in Fig 6.

This process offers several advantages. First, it enables us to select the outstanding prediction method with models from a pool of diverse machine learning methods. As the dominant machine learning methodologies differ based on the specific prediction target, it is crucial to carefully consider and choose the most suitable methodology between various candidates. Second, by comparing the results of the SHAP framework for the selected machine learning methodologies, we enhance the robustness of our findings. This comparative analysis provides additional insights into the contribution of economic indicators and commodities, strengthening the overall validity of our study.

### Machine learning methods to predict BDI

In this section, we will introduce the machine learning methodology that showed the best performance among those used in our research, as well as the central methodology of the entire paper.

#### Extra trees.

Extra Trees is an ensemble supervised machine learning method that constructs multiple decision trees using randomly selected samples without replacement [24]. It closely resembles the RF algorithm but differs in key aspects. Each tree randomly selects a subset of features, and instead of computing an optimal split using entropy, it randomly picks a split value. This leads to diverse and uncorrelated trees.

Thus, Extra Trees builds an ensemble of decision or regression trees using a top-down approach. It differs from other tree-based ensemble methods by randomly selecting split points and utilizing the entire dataset for training, rather than relying on bootstrap replicates. The simplified definition of Extra Trees can be expressed as follows:

$$Y_{\text{pred}}(x)=\frac{1}{M}\sum_{m=1}^{M}f_{m}(x)$$ (1)

where (*x*_*i*_, *y*_*i*_) represents the training set of the model Y, M refers to the total number of decision trees, and *f*_*m*_(*x*) represents the tree that will be trained.

Extra Trees have demonstrated high predictive performance and efficiency in various studies. [25] used Extra Trees for solar thermal energy forecasting, showing competitive performance with Random Forests while being more computationally efficient. [26] applied it to real-time air quality forecasting, effectively capturing high ozone peaks. [27] utilized Extra Trees for stock market return prediction, reducing overfitting and achieving 86.1% accuracy, outperforming Random Forests.

#### CatBoost.

CatBoost is a high-performance, open-source gradient boosting library for classification, regression, and ranking tasks [28,29]. It employs techniques like ordered boosting, random permutations, and gradient-based optimization to enhance performance, particularly on complex datasets with categorical features. By computing the negative gradient of the loss function at each iteration and updating predictions with a carefully selected scaling factor, CatBoost improves precision and efficiency. Additionally, its ordered boosting method optimizes the learning process through deliberate feature permutations, leading to faster convergence and improved accuracy, especially for high-dimensional datasets. The definition of the CatBoost regressor can be expressed as follows:

$$L(f)=\sum_{i=1}^{N}l(y_{i},f(x_{i}))+\sum_{k=1}^{K}\Omega (f^{\left(k\right)})$$ (2)

where *x*_*i*_ is the feature vector of the training set, *y*_*i*_ is the target variable, N is the total number of training samples, K is the number of ensemble trees, $l(y_{i},f(x_{i}))$ is the loss function between the target *y*_*i*_ and the prediction *f*(*x*_*i*_), and $\Omega (f^{\left(k\right)})$ represents the penalty for overfitting of individual trees.

CatBoost has demonstrated high predictive performance and interpretability across various domains. [30] applied it to corporate bankruptcy prediction, accurately identifying firms under financial distress and key risk factors for investors. [31] utilized CatBoost to analyze FCoV spike protein gene mutations, developing an early diagnostic model for feline infectious peritonitis (FIP) with improved survival rates. These studies underscore CatBoost’s predictive power and interpretability in financial risk management and disease diagnosis.

#### Random forest.

Random Forest(RF) comprises a collection of tree predictors, where the construction of each tree relies on a random vector sampled independently, sharing an identical distribution across all trees within the forest [32]. RFs designed for regression involve the development of trees based on a random vector θ, where the tree predictor h(x,θ) yields numerical values rather than class labels. The resulting output values are numerical, and we presume that the training set is drawn independently from the distribution of the random vector (*Y*,*X*). [33]) The definition for any numerical predictor *h*(*x*) can be expressed as follows:

$$h(x)=\frac{1}{B}\sum_{b=1}^{B}h(x,L_{b})$$ (3)

where B represents the number of decision trees, (*x*_*i*_, *y*_*i*_) represents the training set, and *L*_*b*_ represents the tree that will be trained.

RF has demonstrated high predictive performance and efficiency in various studies. [34] used RF to forecast Japan’s real GDP growth from 2001 to 2018, achieving higher accuracy compared to forecasts by the IMF and BOJ. [35] employed RF to create a flood susceptibility map for Ibaraki Prefecture in Japan, achieving an area under the ROC curve (AUC) of 99.56%. [36] improved the RF model to predict financial distress in electronic manufacturing enterprises by applying pruning and SMOTE techniques, achieving an accuracy of 98.03%, thereby supporting sustainable business decisions. These studies highlight the robust predictive capability and practical applicability of RF across different fields.

### SHAP

SHAP is an explainable model that satisfies the properties of additive-feature-attribution methods: local accuracy, missingness, and consistency. It uses Shapley values to fairly attribute contributions to individual features in a prediction [4]. Derived from game theory, Shapley values allocate overall benefit based on the weighted sum of marginal contributions [37]. SHAP represents the conditional probability of Shapley values and assigns importance to each feature for a given prediction. In our study, we apply SHAP to a financial dataset to analyze feature contributions in forecasting BDI.

The Shapley values from game theory are used to attribute $\phi_{i}$ values to each feature in the prediction, as follows:

$$\phi_{i}(f,x)=\sum_{z^{\prime }\subseteq x^{\prime }}\frac{|z^{\prime }|!(M-|z^{\prime }|-1)!}{M!}[f_{x}(z^{\prime })-f_{x}(z^{\prime }\backslash i)]$$ (4)

where $\left|z^{\prime }\right|$ is the number of non-zero entries in $z^{\prime }$, and $z^{\prime }\subseteq x^{\prime }$ represents all $z^{\prime }$ vectors where the non-zero entries are a subset of the non-zero entries in $x^{\prime }$; *M* is the set of all features, $f_{x}(z^{\prime })$ is a trained model with input parameter $z^{\prime }$, and $f_{x}(z^{\prime }\backslash i)$ is a model excluding $z^{\prime }$

$$g(z^{\prime })=\phi_{0}+\sum_{i=1}^{P}\phi_{i}z_{i}^{\prime }$$ (5)

where $z^{\prime }\in \{0,1{\}}^{d}$, *d* is the number of simplified features. Here, $\phi_{i}$ denotes the Shapley value obtained from (5). $\phi_{0}$ is a constant when all the inputs are missing.

[4] introduced SHAP, which is a unified framework for explaining the predictions of complex models, such as XGBoost. The SHAP framework is also independent of complex models and can be easily accessible to them.

SHAP is widely applied beyond predictive modeling. [38] used it to evaluate the impact of features like traffic and population on road accident occurrences. [39] applied SHAP in anomaly detection, identifying key factors influencing outliers. [40] leveraged SHAP for oil price and volatility forecasting by incorporating financial, economic, and political indicators, improving predictive insights.

## Results and discussion

In this section, we present the BDI prediction results obtained through the application of several machine learning models. Additionally, we employ the SHAP framework to interpret the forecasting results produced by these models. The forecasting and SHAP application follow the workflow outlined in Fig 6. Finally, we discuss the empirical findings derived from these analyses.

### The results of BDI prediction and SHAP framework

We begin by predicting BDI using various machine learning methodologies and evaluating their prediction performance. Table 2 presents the performance measures, including Mean Absolute Error (MAE), Mean Squared Error (MSE), Root Mean Squared Error (RMSE), and ${\text{R}}^{2}$ of the models. Based on these performance results, we selected the top three models: Extra Trees, CatBoost, and Random Forest.

**Table 2:** Error measures by different machine learning models.

Model	MAE	MSE	RMSE	${\text{R}}^{2}$
Extra Trees	182.53	73960.07	260.98	0.88
CatBoost	217.45	113153.71	309.86	0.84
Random Forest	238.08	143601.06	349.77	0.79
Light Gradient Boosting	275.33	161889.81	383.88	0.74
Decision Tree Regressor	265.18	163617.62	389.80	0.76
Gradient Boosting	249.28	165981.41	370.36	0.75
Extreme Gradient Boosting	266.20	211568.35	411.07	0.70
AdaBoost	347.35	269155.35	466.75	0.64
K Neighbors	403.74	342622.35	567.89	0.47
Elastic Net	450.11	348666.35	579.08	0.47

Among the ten tested models, Extra Trees achieved the best performance with the lowest MAE (182.53), MSE (73,960.07), and RMSE (260.98), along with the highest ${\text{R}}^{2}$ value of 0.88. CatBoost and Random Forest followed, with MAE values of 217.45 and 238.08, and ${\text{R}}^{2}$ values of 0.84 and 0.79, respectively. These three models significantly outperformed other machine learning methods such as LightGBM, XGBoost, and AdaBoost, which exhibited higher error values and lower predictive accuracy.

Comparing Extra Trees with the weakest performing models, the MAE of Extra Trees was 59.4% lower than Elastic Net (450.11) and 54.8% lower than K Neighbors (403.74), demonstrating its superior predictive precision. Similarly, Extra Trees reduced the RMSE by 54.9% compared to Elastic Net (579.08) and 54.0% compared to K Neighbors (567.89). In terms of ${\text{R}}^{2}$, Extra Trees achieved 0.88, which is 87.2% higher than Elastic Net and K Neighbors, both of which had an ${\text{R}}^{2}$ of just 0.47.

Among the top three models, Extra Trees showed a 16.1% lower MAE than CatBoost (217.45) and a 23.3% lower MAE than Random Forest (238.08). Additionally, Extra Trees achieved a 15.8% lower RMSE than CatBoost (309.86) and a 25.4% lower RMSE than Random Forest (349.77). This further highlights the strong performance of Extra Trees in reducing prediction error.

While these numerical comparisons clearly demonstrate the superior performance of Extra Trees, a deeper analysis is required to understand the underlying reasons behind these differences.

Extra Trees employs significant randomness in both feature selection and split-point determination [24]. This mechanism allows the model to explore a wider range of feature combinations, capturing complex non-linear interactions and reducing overfitting. SHAP analysis supports this behavior by revealing that, although Extra Trees emphasizes critical predictors—such as the S&P 500—it distributes the feature contributions in a balanced yet focused manner [4]. Notably, an increase in the S&P 500 is shown to have a significant impact on the rise of the BDI, suggesting that this predictor is a strong signal that Extra Trees effectively leverages. In contrast, the VIX exhibits a relatively lower SHAP value and functions as a lagging indicator of the S&P 500. Its delayed response implies that, while financial market uncertainty (as captured by the VIX) does affect the BDI, its impact is less immediate.

In comparison, CatBoost, which is built on gradient boosting and handles categorical variables effectively [29], exhibits a more uniform distribution of feature importance. The SHAP plots for CatBoost indicate that contributions from various predictors are more evenly spread out, resulting in stable predictions. However, this even distribution can lead to a diluted emphasis on the most potent predictors, which may explain why CatBoost shows slightly higher errors than Extra Trees.

Random Forest, which aggregates predictions from multiple trees using bagging, shows a tendency to rely heavily on a few dominant features as revealed by its SHAP analysis [32]. This concentrated reliance on select predictors, such as the immediate effects of the S&P 500, can make Random Forest more sensitive to fluctuations in these key features, potentially resulting in higher variability and error compared to the more balanced approach of Extra Trees.

Furthermore, models with poorer performance—such as AdaBoost, K Neighbors, and Elastic Net—likely suffer from limitations in capturing complex, non-linear interactions and are more sensitive to noise or overfitting. Their higher error metrics suggest that these models may not effectively leverage key predictors like the S&P 500, which, as the SHAP analysis demonstrates, plays a crucial role in forecasting the BDI.

Additionally, previous research (e.g., [50]) has observed that an increase in the VIX tends to lead to a decline in the BDI, indicating that rising financial market uncertainty adversely affects the shipping market. This economic insight reinforces the interpretation that predictors like the S&P 500, which show a strong and immediate effect on the BDI, are essential for accurate forecasting. Extra Trees appears to capitalize on these strong signals more effectively than both CatBoost and Random Forest.

In summary, the superior performance of Extra Trees is not solely reflected in its lower MAE and RMSE but is fundamentally rooted in its ability to harness complex feature interactions and focus on key predictors, as validated by SHAP analysis. This integrated approach—linking model mechanics, numerical performance, and economic context—offers a comprehensive explanation of why Extra Trees outperforms the other models and clarifies the nuanced differences between CatBoost and Random Forest, as well as the reasons behind the poorer performance of models such as AdaBoost, K Neighbors, and Elastic Net.

We conducted additional fine-tuning using these top three models. The results confirmed that Extra Trees demonstrated the highest performance, followed by CatBoost and Random Forest. Additionally, they all shared common contributors, such as S&P 500, DXY, and coal. Notably, S&P 500 was consistently ranked as the top contributor for weekly data generated by all models. These results enhance the robustness of our study.

**Fig 7:**
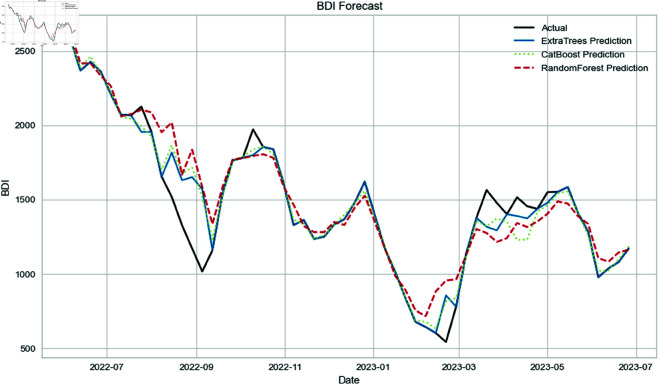
The weekly BDI index prediction generated by Extra Trees, CatBoost, and Random Forest.

Fig 7 illustrates summary plots of BDI predictions using the Extra Trees, CatBoost, and Random Forest models. All the participating models demonstrated good performance; however, the XGBoost model exhibited the lowest prediction error. On the contrary, the RF model showed the highest prediction error, especially from August 2022 to September 2022 and February 2023 to May 2023. Extra Trees also performed well, but the XGBoost model was slightly better in certain details, such as in August 2022 and March 2023. LightGBM showed quality in predictions after September 2022 but exhibited a notable divergence from the actual BDI index trend.

We apply the SHAP framework to the prediction results of each simulation. The outcomes of applying the SHAP framework to each model are detailed below.

**Fig 8:**
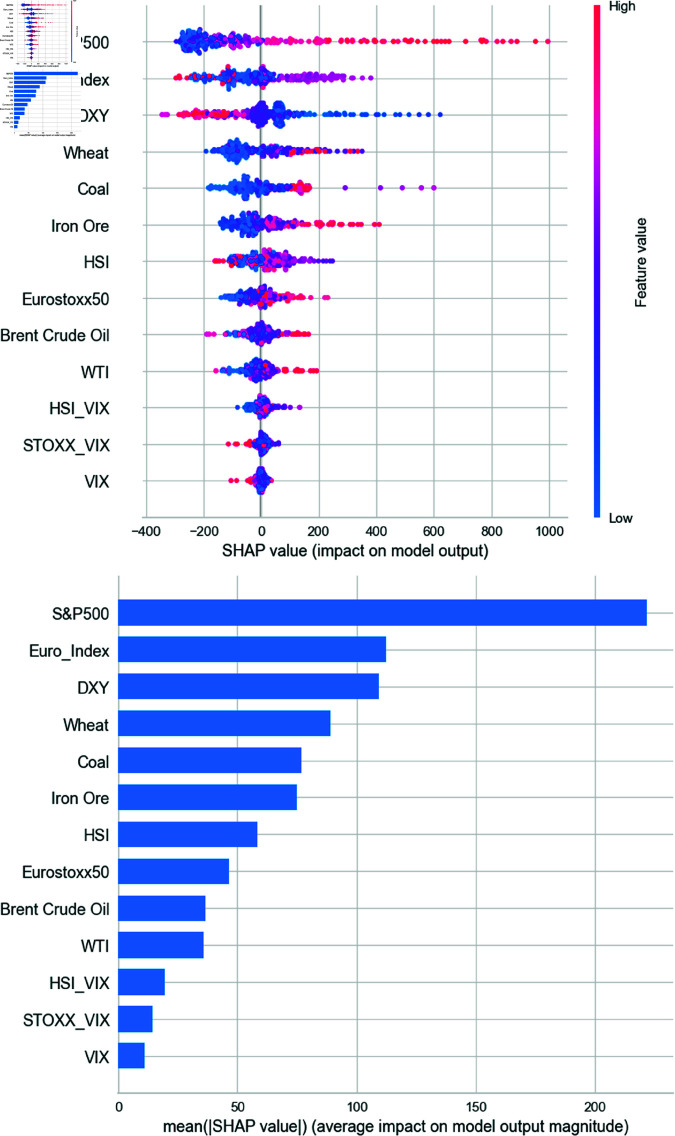
SHAP results from Extra Trees regressor model.

Fig 8 presents the SHAP summary plot using the Extra Trees regressor. A positive SHAP value indicates an increase in the predicted BDI relative to the baseline, while a negative value implies a decrease. Red dots on the positive (negative) side denote that higher feature values are positively (negatively) correlated with the prediction. Conversely, blue dots on the positive side suggest that lower feature values increase the prediction. The plot reveals that the S&P 500 has a strong positive contribution, while the DXY and Euro index exhibit negative correlations. Commodities also contribute meaningfully, whereas volatility indices show minimal impact.

**Fig 9:**
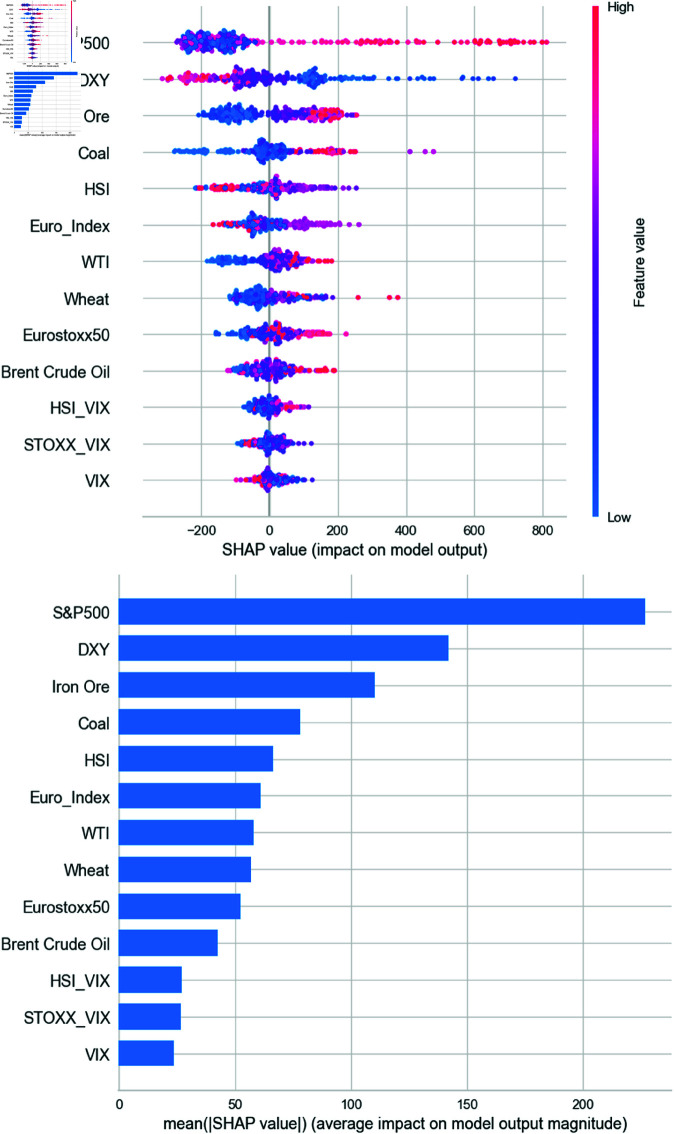
SHAP results from CatBoost regressor model.

Fig 9 shows the SHAP summary using the CatBoost regressor. Consistent with Extra Trees, the S&P 500 demonstrates the highest positive impact, followed by DXY with a negative contribution. Notably, CatBoost indicates a strong positive influence of iron ore when SHAP values exceed 100. Coal also emerges as an important contributor, while other features, including additional commodities and stock indices, have lesser effects. Volatility indices again exhibit negligible influence.

**Fig 10:**
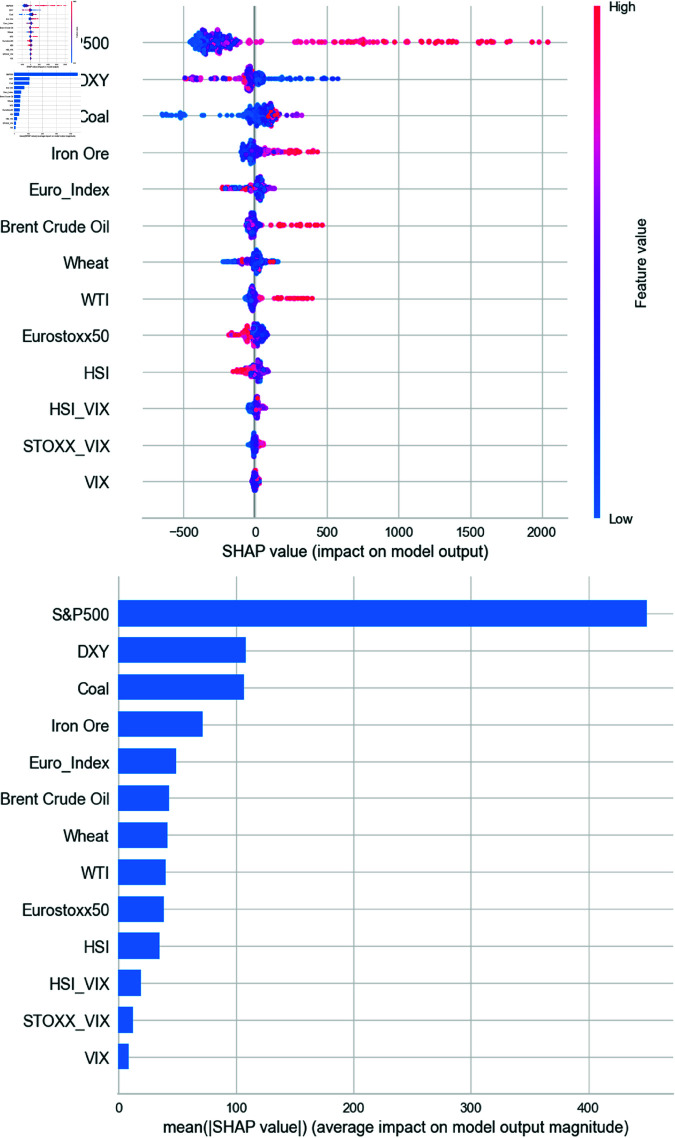
SHAP results from Random Forest regressor model.

Fig 10 displays SHAP values from the Random Forest model. Similar patterns are observed: the S&P 500 leads in influence, followed by commodities such as coal, iron ore, and crude oil, all positively correlated with BDI. Currency and commodity variables play crucial roles, while volatility indices consistently show minimal explanatory power, raising questions about their relevance in predicting BDI.

We conducted our experiments using models for weekly data, including the Extra Trees regressor, CatBoost regressor, and RF. According to Figs 8 to 10, we confirmed through the SHAP analysis that the S&P 500, DXY, iron ore, and coal significantly contribute to the prediction of BDI. The findings for each asset are interpreted in relation to previous literature as follows.

First, one of the key findings from the SHAP analysis is that the S&P 500 index exerts the strongest influence on BDI. In all three models, SHAP plots consistently show that increases in the S&P 500 lead to increases in the BDI, while decreases in the index correspond to declines in the BDI. Overall, the SHAP analysis identifies the S&P 500 as the most important variable in predicting BDI.

This outcome can be interpreted through the economic mechanism linking financial markets with the maritime industry. A strong performance in the S&P 500 typically reflects heightened investor confidence and corporate expansion, which leads to increased production and demand for shipping services, thereby boosting BDI. Conversely, a downturn in the stock market suggests reduced investment and slower trade, exerting downward pressure on freight rates and BDI. Furthermore, the S&P 500 can be seen as a leading indicator that anticipates global economic signals, including investor sentiment, economic growth, and corporate performance. According to [51], the S&P 500 influences investor behavior, corporate capital expenditure, and commodity demand, all of which directly affect maritime freight volumes and rates. In addition, [52] highlights the close connection between financial markets and the shipping industry, noting that the latter exhibits high systematic risk due to financial and operational leverage.

Several previous studies have already demonstrated the role of BDI as a global economic indicator in relation to the S&P 500. For example, [41] employed the S&P 500 bank index as a proxy for U.S. bank performance and BDI as a proxy for global economic activity to analyze how BDI and other economic indicators affect U.S. bank indices. Similarly, [42] examined the impact of the COVID-19 pandemic on the S&P 500 by treating both the S&P 500 and BDI as indicators of global economic conditions, emphasizing the relevance of global supply and demand in shaping BDI. Notably, these studies emphasized the strong relationship between BDI and equity returns, particularly in terms of variance. Such findings align with our SHAP analysis results, which also underscore the significant role of the S&P 500 in BDI prediction.

Second, among currencies, the DXY plays a critical role in predicting BDI. DXY is widely recognized as a key macroeconomic indicator used in forecasting BDI and various other economic variables. Numerous studies have used DXY as an economic proxy to predict BDI or examine the financial relationship between the two. For instance, [11] emphasized the importance of DXY in forecasting BDI, and [14] treated BDI as a key indicator in exchange rate predictions, including DXY. [13] analyzed the cyclicality of the BDI and proposed hedging strategies by identifying both positive and negative correlations with DXY. In particular, their study highlighted a negative correlation, suggesting that a weaker dollar is often associated with stronger global economic growth, especially in emerging markets, which in turn boosts the BDI. This is consistent with the findings of our SHAP analysis.

Third, among commodities, iron ore and coal significantly contribute to BDI prediction. Iron ore and coal account for approximately 27% and 26% of total dry bulk shipping volume, respectively, making them essential drivers of the BDI. The SHAP analysis demonstrates that rising prices of these commodities reflect increased industrial demand and are associated with higher BDI values. Conversely, falling prices indicate reduced production and lower demand for maritime transportation, resulting in a decrease in BDI. This bidirectional relationship is consistent with previous studies such as [16] and [43], which recognize the BDI as a key indicator for assessing the supply, demand, and usage of core global commodities.

In summary, both previous literature and our SHAP analysis confirm that major macroeconomic indicators—DXY, the S&P 500, and commodity prices—play a vital role in explaining BDI fluctuations. A weaker dollar typically signals stronger global economic growth and trade, leading to increased shipping demand and higher BDI, while a stronger dollar reflects slower economic activity and reduced shipping volumes. Similarly, a rising S&P 500 is associated with business expansion and trade growth, driving up BDI, whereas a declining index indicates contraction and reduced freight volumes. With regard to iron ore and coal, rising prices suggest strong industrial output and higher transportation demand, while declining prices imply weaker economic activity. These findings collectively underscore the interconnected influence of currency markets, equity indices, and commodity prices in explaining the dynamics of BDI and the global shipping industry.

These findings offer several noteworthy implications. First, financial assets such as the S&P 500 and DXY make substantial contributions to BDI prediction. This finding is supported by the widespread recognition of the BDI as a global economic indicator. For instance, [44] and [45] evaluated BDI as a leading indicator of economic growth based on dry cargo ocean freight rates and global economic activity. Additionally, [46] investigated the relationship between BDI and U.S. economic growth, while [11] considered 42 potential global economic and BDI-related variables to improve prediction accuracy. These studies collectively suggest that both the BDI and S&P 500 reflect broader global economic trends.

Such a relationship becomes markedly pronounced during specific economic events. For example, [53] report that following the WHO’s pandemic declaration on March 11, 2020, the BDI exhibited a delayed decline four to five days later, suggesting a temporal spillover from the initial downturn in the financial market (S&P 500) to the shipping sector. Similarly, [3] find that during the 2008 subprime crisis, the BDI transmitted volatility to both the U.S. Dollar Index (DXY) and the Global Commodity Index (GSCI), emphasizing its role in amplifying market stress. [54] further show that financial market fluctuations influence shipping contract preferences: firms favor spot markets during off-peak uncertainty and shift to long-term contracts in peak periods. These findings highlight the broader impact of financial instability on the structural dynamics of global shipping markets.

DXY also showed a meaningful contribution to BDI forecasting. The negative correlation between DXY and BDI suggests that a stronger U.S. dollar tends to dampen global trade activity, thereby reducing the demand for dry bulk shipping. This is consistent with the findings of [13], who identified a strong negative relationship between DXY and BDI and used this relationship to explore BDI cyclicality and hedging strategies. Our results align with this literature, indicating that BDI and DXY generally move in opposite directions—when DXY declines, BDI tends to rise.

Second, commodity assets also play a key role in predicting BDI. Since BDI is closely linked to bulk carrier capacity, commodity flows, oil prices, and shipping routes, it is unsurprising that major dry commodities such as wheat, coal, and iron ore exert a positive influence on BDI. When the prices of these commodities increase, BDI tends to rise as well. This finding is particularly important given that these commodities represent significant volumes in the dry bulk shipping sector. [13] reported that iron ore and coal alone account for over 50% of global dry commodity trade. Furthermore, [16] examined asymmetric causality between BDI and commodities, concluding that iron ore and coal exhibit a stable bidirectional relationship with BDI. Other studies, such as [1,13], have also adopted commodity prices as sub-indicators for forecasting BDI. These findings support the strong link between BDI and commodity prices, which is consistent with our result that BDI exhibits a symmetric trend with commodity price increases and decreases.

### Discussion

According to the SHAP framework results of the selected models, we found that some variables consistently ranked high in their contribution to BDI. Two results were significant; one was expected, but the other one was surprising. First, there was an intrinsic relationship between BDI, iron ore, and coal, which explains the excellent predictive performance of the model. It is widely recognized that iron ore and coal are crucial components of dry bulk commodities due to their significant roles in industrial processes and global trade. Second, unexpectedly, S&P 500 demonstrated the most pronounced influence in all models, with the DXY index ranking as the second most influential factor. Other variables, including currency exchange rates, stock indices (except for the S&P 500), and the remaining commodities, exhibited analogous behaviors. Moreover, the volatility indices displayed negligible significance in their contribution to BDI. Hence, the second result underscores the imperative of explaining the relationship between BDI and the US economy within the investigation.

According to several studies, BDI and the US economy showed an indirect relationship. [41] examined the relationship between BDI and US bank indices (Dow Jones, S&P 500) using the wavelet coherence method. They discovered that the relationship between BDI and US bank indices was inconsistent; inconsistencies were observed in the lead-lag association. In [47], the interconnections between BDI, S&P 500, and Dow30 became less pronounced, particularly following the subprime mortgage crisis. These findings could provide valuable insights for participants in the dry bulk shipping market to enhance their decision-making processes. Moreover, [48] claimed that the assertion of the BDI serving as a leading economic indicator for the US economy in relation to GDP in USD remains subject to uncertainty. The findings underscored the necessity of employing diverse methodologies to establish a substantiated and significant connection between the BDI and GDP in USD.

One of the notable outcomes of our study pertained to the unanticipated influence of S&P 500 on BDI. This discovery contrasts with our initial expectations and merits examination. Notably, the calculation formula for BDI encompasses components such as the BCI, BPI, and BSI, distinguished by bulk carrier size and distinct maritime routes. Furthermore, the pricing of crude oil assumes a pivotal role in determining BDI fluctuations. Within this framework, several facets may illuminate the potential reasons for the impact of the US economy on BDI. Initially, the price standard of dry cargo has a deep relationship with the US economy. Second, crude oil prices (Brent, WTI) are affected by the US stock market. Last, various major US companies are involved in the shipping industry (Union Pacific Corp, Royal Caribbean Cruises Ltd, Carnival Corp, Huntington Ingalls Industries, and Norwegian Cruise Line Holding), and trade contracts are followed. Furthermore, [48] argued that the claim of BDI being a leading economic indicator for the U.S. economy, particularly concerning GDP in USD, remains inconclusive. Their findings highlighted the need for employing diverse methodologies to establish a robust and meaningful correlation between the BDI and GDP in USD.

From a logistical perspective, the strong relationship between the U.S. economy, iron ore, coal, and the BDI provides valuable insights. As the BDI is regarded as a crucial index of global trade and the shipping industry, several logistical conclusions can be drawn.

First, iron ore and coal are significant commodities in the U.S. as they are directly related to the manufacturing and energy industry. If the supply of these two commodities is efficient, the industry will be more active. Second, BDI usually affects marine transportation. The cost of transportation rises when the BDI increases, directly impacting the global trade of goods and commodities. To remain competitive in the international market, U.S.-produced iron ore and coal must manage logistics costs. The fluctuation in the BDI affects the logistical network and supply chain in the U.S., as well as in other countries. Iron ore and coal are purchased in the global market, especially during unstable BDI or world economy circumstances, which can affect logistical activities between the U.S. and other countries. This correlation provides important information for optimizing and making global logistical networks more efficient.

This study innovatively incorporates the volatility index as a predictive variable for the BDI, diverging from previous research in this field. However, SHAP analysis reveals that the impact of the volatility index on BDI prediction is relatively minor compared to other key variables. These findings offer several important implications.

First, the results suggest that BDI forecasts are more strongly influenced by real economic downturn indicators—such as a decline in the S&P 500 index—than by measures of financial market uncertainty like the VIX. Second, the VIX primarily reflects short-term investor sentiment and expected volatility in the U.S. equity market. In contrast, the BDI tends to respond to broader macroeconomic conditions and longer-term economic trends rather than short-term fluctuations in financial markets. Finally, the relatively low SHAP value of the VIX may be attributed to lagged or indirect effects. For instance, during periods of heightened market volatility, trade volumes may contract due to increased investor caution or tightened credit conditions. However, such effects are unlikely to be immediately reflected in dry bulk shipping demand or may be overshadowed by more direct determinants such as commodity prices and macroeconomic indicators. These factors help to explain why the VIX contributes less significantly to BDI prediction in the empirical models presented in this study.

## Summary and concluding remarks

In this study, we predict the BDI using various machine learning methods and examine the financial features that contribute to BDI prediction based on the SHAP framework. Rather than relying on a single model, we support our findings by comparing three top-performing models and identifying overlapping values among the 13 selected factors.

The results generated using the SHAP framework highlighted several critical factors influencing the prediction of the BDI. First, the S&P 500, used as a proxy for U.S. economic indicators, demonstrated a significant influence across all models. This consistent dominance underscores the critical role of the U.S. economy in predicting the BDI, aligning with the findings of [22].

Second, commodity prices emerged as another significant factor, emphasizing the fundamental link between the BDI and key commodities. In particular, iron ore and coal, essential dry goods for the energy and metals sectors, were identified as major contributors. This result aligns with [16], who also established a strong relationship between these commodities and the BDI.

Third, the dollar index played a crucial role in BDI prediction. Along with the S&P 500, the contribution of the dollar index highlights the interconnectedness of the U.S. economy and the BDI. This finding is further supported by [3], who demonstrated the relationship between the dollar index (DXY) and the BDI.

Lastly, various machine learning algorithms were applied, with Extra Trees, CatBoost, and Random Forest demonstrating excellent performance and efficiency in error measurement, validating the research results. Additionally, this study utilized the SHAP framework to identify key factors affecting the BDI, a method that distinguishes it from previous BDI prediction studies.

These results offer significant implications for stakeholders in the shipping industry. First, BDI forecasting is a practical concern for these stakeholders, and this study provides a robust framework that demonstrably enhances BDI prediction capabilities. Second, we analyzed the influence of various indicators on BDI prediction, offering valuable considerations for decision-makers. For instance, given the influence of the S&P 500 demonstrated in this study, it is crucial to consider not only traditional factors such as product prices and the type and size of ships but also the economic situation in the United States when predicting BDI.

The findings of this study not only provide meaningful insights into BDI prediction but also contribute to enhancing the stability of the shipping market. Improving market transparency, upgrading infrastructure, and adopting data-driven decision-making are essential for strengthening overall market resilience.

Specifically, the study offers practical implications for strategic decision-making in the shipping industry. The predictive power of financial indicators such as the S&P 500 can be directly applied by market participants to optimize freight rate forecasting and hedging strategies. For instance, shipowners and charterers engaging in Forward Freight Agreements (FFAs) can monitor fluctuations in the S&P 500 and commodity prices to anticipate spot market volatility. [57] empirically confirmed that the S&P 500 Commodity Index and oil prices significantly influence volatility in the Capesize freight market. Their research also showed that freight derivatives markets like FFAs incorporate macroeconomic signals more rapidly than the physical market, allowing participants to form better-informed expectations.

Moreover, standardizing indices and utilizing real-time data can improve understanding of the relationship between the BDI and equity markets, enabling stakeholders to make more informed decisions and reduce price volatility [3]. Expanding port infrastructure and implementing digital transformation are also critical for enhancing demand forecasting accuracy, improving operational efficiency, and managing the complex temporal dynamics of the shipping industry [49].

Diversifying trade routes and establishing alternative shipping hubs can reduce dependence on congested lanes and improve the resilience of global supply chains. Finally, promoting financial hedging mechanisms offers stakeholders effective tools to manage price fluctuations, fostering a more stable and predictable shipping market [16].

Our research has several limitations. For example, we did not extensively incorporate intuitive information that directly contributes to BDI, such as cargo volumes, adverse weather conditions, and shipping routes. Since the BDI reflects the cost of maritime transportation for raw materials such as iron ore, coal, and grain, the volume of these key cargoes is directly linked to freight demand. Therefore, incorporating cargo volume data into BDI forecasting models is expected to enhance their predictive accuracy. In addition, weather conditions are a critical factor influencing maritime logistics, affecting sailing speed, port operations, and the availability of shipping routes. As such, weather data may also have a meaningful impact on the accuracy and reliability of BDI predictions. Therefore, we propose the development of BDI forecasting models that incorporate these additional factors as a direction for future research.

Furthermore, incorporating real-time satellite data on maritime traffic, port congestion, and vessel movement could significantly refine BDI predictions. Advanced remote sensing techniques, such as Synthetic Aperture Radar (SAR) and Automatic Identification System (AIS) data, can provide insights into fleet utilization, route congestion, and fuel consumption trends. Future research could explore how deep learning models can process and integrate such data to improve forecast precision. Moreover, analyzing the influence of environmental policy variables could be considerable. With increasing regulatory focus on emissions and sustainability, environmental policies have begun to affect shipping costs and fleet availability. Research can focus on quantifying the impact of regulations such as the International Maritime Organization’s (IMO) carbon intensity targets or regional emission control areas (ECAs) on BDI trends. Machine learning models that incorporate policy shifts and compliance costs could offer a more comprehensive approach to forecasting.

Additionally, beyond the adoption of S&P 500 and DXY as US economic indicators, we did not use other comprehensive indicators of the US economy. These limitations suggest possibilities for consideration in future research.
